# Up-regulation of anti-apoptotic genes confers resistance to the novel anti-leukaemic compound PEP005 in primary AML cells

**DOI:** 10.18632/oncoscience.71

**Published:** 2014-08-06

**Authors:** Peter Hampson, Keqing Wang, Elisabeth Ersvær, Emmet McCormack, Julia Schüler, Heinz-Herbert Fiebig, Bjørn Tore Gjertsen, Øystein Bruserud, Janet M. Lord

**Affiliations:** ^1^ School of Immunity and Infection, University of Birmingham, Birmingham, B15 2TT, UK; ^2^ Department of Biosciences, University of Aston, Birmingham, B4 7ET; ^3^ Institute of Medicine, University of Bergen, Bergen, NO-5020, Norway; ^4^ Oncotest GmbH, 79801 Freiburg, Germany

**Keywords:** AML, PEP005, PKC, anti-apoptotic, xenograft model

## Abstract

We showed previously that PEP005 induced apoptosis in leukaemic cell lines and blasts from patients with acute myeloid leukaemia (AML). Here we assess the anti-leukeamic effects of PEP005 *in vivo* and determine the mechanism of resistance of PEP005 non-responsive cells. We used 2 human xenograft mouse models of AML to assess the anti-leukaemic effects of PEP005 *in vivo*. Expression microarray analysis of primary AML blasts following treatment with PEP005 was used to determine patterns of gene expression that conferred resistance. PEP005 significantly reduced tumour burden in two human leukaemia mouse xenograft models. We also assessed responsiveness of 33 AML samples to PEP005, with 78% of the samples entering apoptosis at 100nM. Resistance to PEP005 was not restricted to a particular AML subtype. Expression microarray analysis of resistant samples following treatment with PEP005 revealed a significant up regulation of the anti-apoptotic genes Bcl-2A1, Mcl-1, and PHLDA1 which was verified using RT-PCR. We conclude that PEP005 shows broad efficacy against AML subtypes and that up regulation of anti-apoptotic genes underlies resistance to this agent and could be used to screen for patients unlikely to benefit from a therapeutic regime involving PEP005.

## INTRODUCTION

The incidence of Acute Myeloid Leukaemia (AML) increases with age, with a majority of cases occurring in people over 65 [1]. Whilst 40%-65% of patients ≥60 will achieve remission, around 85% will relapse within 2-3 years [2]. This poorer prognosis is attributable in part to the fact that these patients are less responsive to myelosuppressive chemotherapy and are less able to tolerate therapy [3]. Overcoming these problems requires the development of adjunctive therapies that improve tumour responses while not exacerbating the systemic toxicities of established chemotherapeutics.

Extracts from the plant *Euphorbia peplus* (*E. peplus)* are active against a number of tumour cell lines *in vitro*, including strains of malignant melanoma resistant to conventional chemotherapeutic agents [4]. The active ingredient from this plant has been identified as ingenol 3-angelate (PEP005). Topical application of PEP005 was able to cure sub-cutaneous tumours established in C57BL/6 and *Foxn1^nu^* mice [5] and the compound has successfully completed phase IIb trials for non-melanoma skin cancer and solar actinic keratosis [6;7] and came to market for actinic keratoses in 2012.

**Figure 1:**
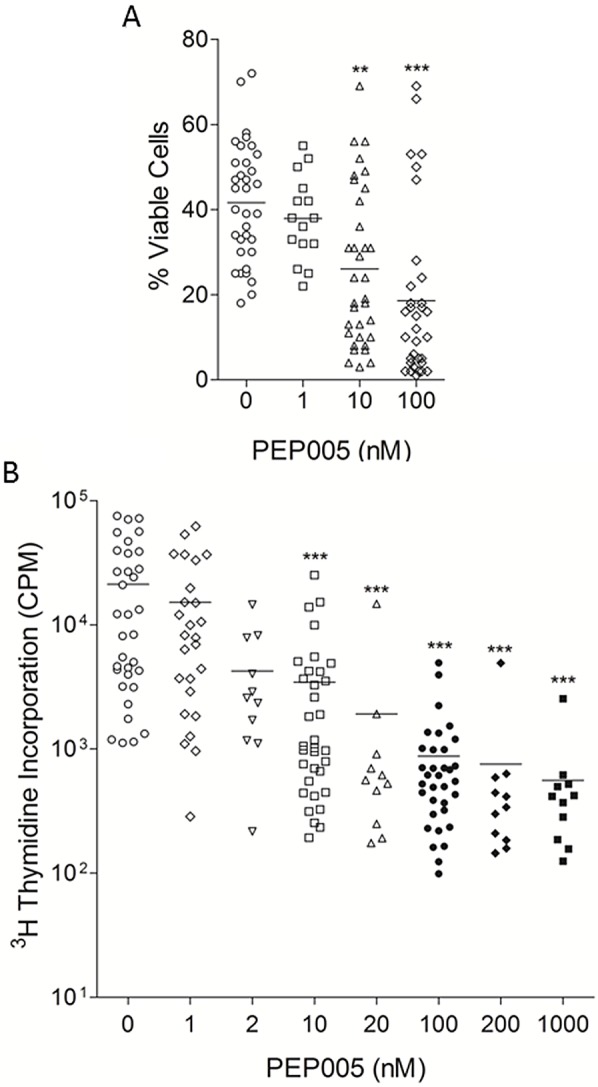
PEP005 has anti-leukaemic effects in vitro Primary AML blasts were isolated from the peripheral blood of patients diagnosed with AML. A. Isolated cells were cultured in the absence or presence of the indicated concentrations of PEP005 for 3 days. Cell viability was measured using annexin-V/PI staining. Annexin-V negative and PI negative cells were taken as the viable cell population. B. Isolated cells were cultured in medium containing SCF, GM-CSF and Flt-3 ligand, in the presence or absence of the indicated concentrations of PEP005. 7 days later, proliferation was measured using 3H-thymidine incorporation and data are expressed as counts per minute (CPM). The horizontal bar indicates the mean value. ** indicates p<0.01, *** indicates p<0.001

Our data have demonstrated that PEP005 induces apoptosis in several leukaemic cell lines [8] and can cause the proliferation of T cells isolated from the peripheral blood of AML patients following intensive chemotherapy [9]. This is important as it has been demonstrated that AML patients who display early lymphoid reconstitution after conventional intensive chemotherapy have a reduced risk of later relapse [10;11]. Furthermore, PEP005 enhances anti-CD3 and anti-CD28-induced cytokine release by these cells [12]. AML is classified into subtypes M0 to M6. As the different AML subtypes have varying pathogenic mechanisms they are treated with distinct therapeutic regimes. Although our previous publication showed that primary AML cells were responsive to and GM-CSF, along with various concentrations of PEP005, and proliferation assessed with a ^3^H-thymidine incorporation assay. PEP005 inhibited the proliferation of AML blasts in a dose-dependent manner (Figure 1B). Inhibition was seen at doses as low as 2 nM, with optimal effects observed at 100 nM and inhibition of cell proliferation by PEP005 was seen in all primary AML samples.

### PEP005 inhibits tumour growth in human AML xenograft mouse models *in vivo*

**Figure 2:**
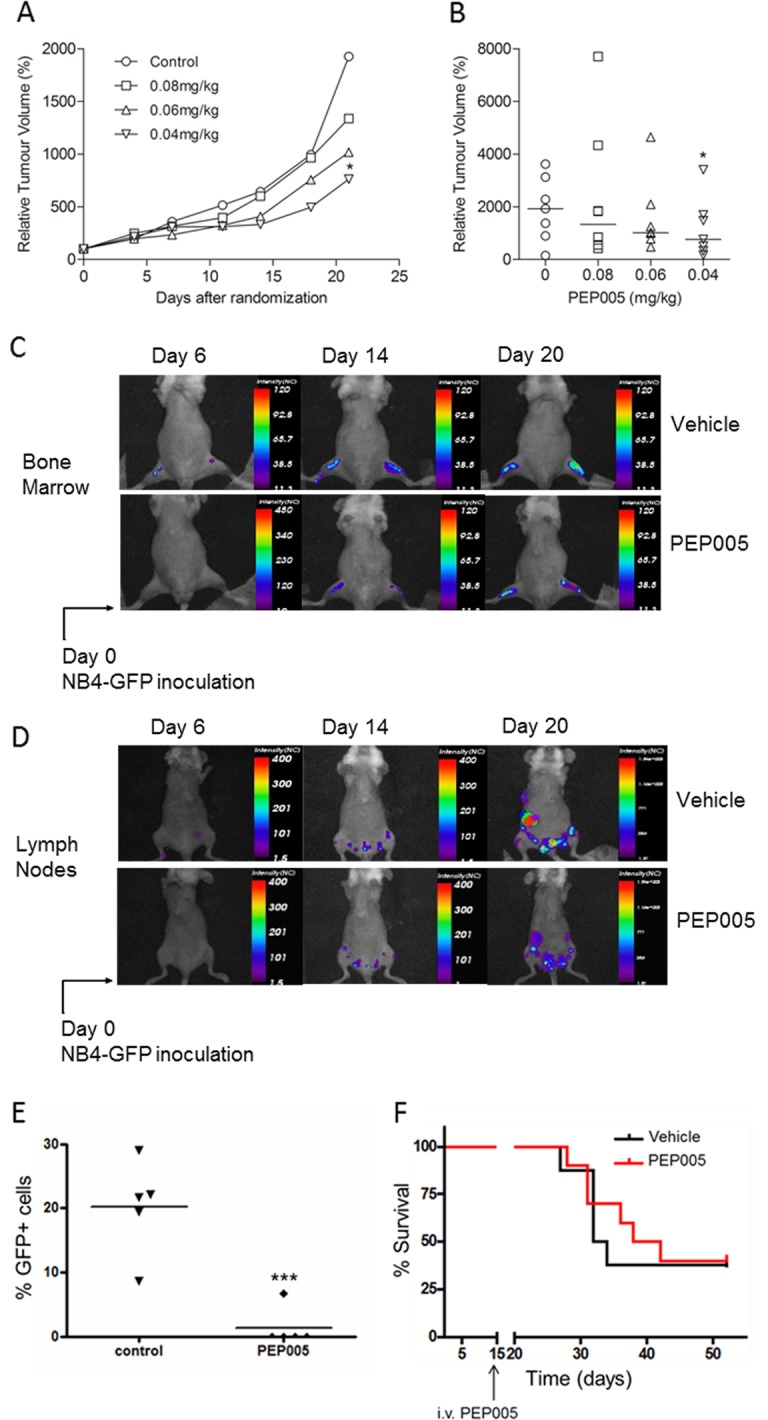
The effect of PEP005 in two human xenograft mouse models of AML U937 cells were injected subcutaneously into nude mice (A and B). Once cells had formed palpable tumours mice were randomized into four groups (8 mice per group) and different doses of PEP005 treatment were initiated. PEP005 was administered to mice by intravenous injection 3 times a week for 2 weeks. A. Tumour volumes were measured twice a week from day 1, and relative tumour volumes were calculated by (tumour size at day x/tumour size at day 0) × 100. Each point represents the median value of relative tumour volumes. B. Data are shown for individual animals at day 21. The horizontal line represents the median value. * indicates p<0.05. (C-F) GFP expressing NB4 cells were intravenously transplanted in to NOD/SCID/β2mnull mice. After 2 weeks inoculation, mice were divided into two groups (6 mice per group). Vehicle control or 50 mg/kg of PEP005 was administered to mice by intravenous injection daily for 5 days. Recipient mice were monitored and sacrificed when moribund, as defined by weight loss, lethargy and/or paralysis. Images showing GFP-NB4 cell engraftment in the bone marrow (C) and lymph nodes (D) following vehicle or PEP005 treatment. Prior to imaging, anaesthetised mice were depilated and moved to the heated translational stage of the eXpore Optix™. Animals were subsequently maintained under gas anaesthesia during scanning. GFP fluorescence images were generated using Optiview™ software. E. Inguinal lymph nodes were collected after mice were sacrificed and GFP positive (GFP+) cells were enumerated by flow cytometry. Data were analyzed by Flowjo software. *** indicates p<0.001 using two-way ANOVA. F. Survival curves in response to either vehicle (black) or PEP005 (red) treatment.

To evaluate the ability of PEP005 to inhibit the growth of human AML cells *in vivo*, we used two distinct human AML xenograft mouse models: a subcutaneous xenograft model and a systemic xenograft model. In the U937 subcutaneous model, tumour size was measured every 3 days after the start of PEP005 treatment. We found that PEP005 induced significant tumour growth inhibition when applied at 40 mg/kg/d. The most impressive tumour growth suppression was induced three weeks after start of treatment (Figure 2A and 2B), with the relative increase in tumour volume in the PEP005 treated group reduced from 1929% to 763.7%.

Although subcutaneous xenograft models of leukaemia and lymphomas have their advantages, the major limitation of this model is reproducible growth only as localised myelosarcomas with little evidence of engraftment in clinically more relevant organ sites [16]. We therefore employed another xenograft model, using NOD/SCID mice as recipients. These mice were intravenously transplanted with GFP expressing NB4 cells, this cell line being chosen as we had previously shown it had good sensitivity to PEP005 [13]. Using this model, we were able to determine the degree of engraftment of leukaemic cells in different tissues by analysing GFP^+^ cell populations. In Table 1, NB4 cell engraftments in NOD/ SCID mice in different tissues are detailed, showing that a significant effect of PEP005 was seen upon the numbers of GFP-NB4 leukaemic cells infiltrating the superficial cervical lymph nodes. In subsequent experiments, NOD/ SCID/β2m^null^ mice were used as recipients as these mice displayed improved engraftment of leukaemic cells. Whole body imaging indicated that engraftment had occurred in the bone marrow (Figure 2C) and also extensively in other organs including the inguinal lymph nodes and ovaries (Figure 2D). Engraftment was visibly reduced at day 14 and day 20 after inoculation in the PEP005 treated animals (Figure 2C and 2D) and there was a significant decrease in GFP-NB4 cell infiltration into the abdominal lymph nodes at time of sacrifice (Figure 2E). These results show a striking anti-leukaemic activity of PEP005 in this aggressive murine model of AML. Despite this, PEP005 treatment did not significantly increase the survival of the mice (Figure 2F), though this could have been due to low sample size.

**Table 1:** Tissues removed from control or PEP005 treated mice after inoculation and engraftment with GFP-NB4 human leukaemia cells were enumerated for GFP positive (+ve) cells by flow cytometry. Data are total cells (× 10^6^) per tissue and are mean ± SD for 6 mice per group

GFP-NB4 Infiltration (total cells ×10^6^ per tissue)							
Treatment	Mice	Liver	Spleen	Bone Marrow	SC Lymph Nodes	Brain	Blood
**Control**	6	1.4 ± 0.75	2.0 ± 0.2	1.2 ± 0.41	20.2 ± 7.24	3.3 ± 1.45	1.5 ± 0.41
**PEP005(50 μg/kg/d)**	6	0.5 ± 0.29	2.4 ± 1.01	0.9 ± 0.46	1.34 ± 3.68p<0.01	4.7 ± 2	1.1 ± 0.46

### AML patient samples can be grouped according to their response to PEP005

**Figure 3:**
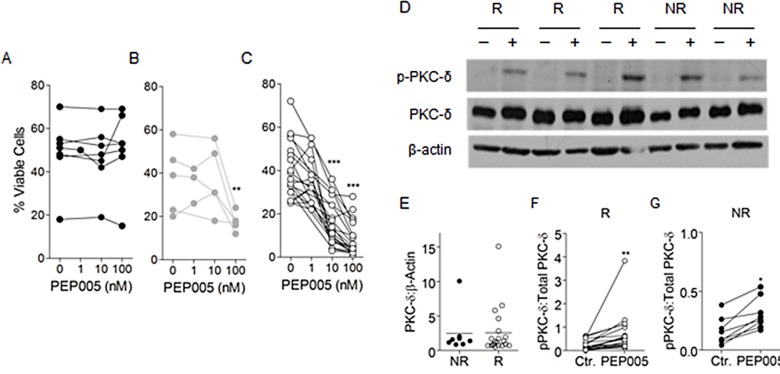
PEP005 treatment leads to the activation of PKC-δ in both responsive and non-responsive patient samples Primary AML samples were grouped into non-responders (A), low-responders (B) or responders (C) based on their apoptotic response to PEP005 treatment. Apoptosis was measured using Annexin V/PI staining. D. Isolated AML blasts were cultured in medium alone (−) or 10 nM PEP005 (+) for 30 minutes. SDS-PAGE and western blotting were then used to measure phospho-PKC-δ and PKC-δ expression, with β-actin used as a loading control. Samples that did not respond to PEP005 treatment in the apoptosis experiments were termed non-responders (NR), and those that did respond were termed responders (R). E–G. Densitometric analysis was used to determine the ratio of total PKC-δ to β-actin (E) and phosphorylated to total PKC-d in both R (F) and NR (G) samples. * indicates p<0.05, ** indicates p<0.01.

Seven of the patient samples tested (21%) had no apoptotic response to PEP005 and were deemed to be non-responders (Figure 3A). 5 of the patient samples (15%) responded to PEP005 only at the relatively high dose of 100 nM and were termed low responders (Figure 3B). The majority of patient samples (21 = 64%) responded to PEP005 at 10 nM and were termed high-responders (Figure 3C). Thus we investigated which cellular factors determined responsiveness of these patient samples to PEP005. Responsiveness was not restricted to any particular AML subtype and Flt-3 and NMP1 mutation status and karyotype did not impact upon sensitivity to PEP005 (Supplementary Table 1).

### The resistance of AML blasts to PEP005 treatment does not relate to PKC-δ expression or activation

We have previously shown in leukaemic cell lines, that responsiveness to PEP005 was dependent on the expression and activation of PKC-δ [13]. We were therefore interested to see whether there was any correlation between PKC-δ expression and PEP005 responsiveness in primary AML blasts. Figure 3D shows an example of the results obtained for five of the patient samples tested (3 PEP005 responsive and 2 PEP005 non-responsive), showing that all of these patient samples expressed high levels of PKC-δ. Densitometric analysis on 28 patient samples (20 PEP005 responsive and 8 PEP005 non-responsive), measuring PKC-δ expression as a ratio to the expression of β-actin revealed no significant difference in PKC-δ expression between these samples (Figure 3E).

PKC-δ activation was measured by detecting the phosphorylated active form of this PKC isoenzyme. Leukaemic PBMCs were treated for 30 minutes with 10 nM PEP005. Phosphorylation of PKC-δ was then measured by western blotting using an antibody specific for PKC-δ phosphorylated at threonine 505 (Thr^505^), with expression of total PKC-δ used as a loading control. Figure 3D shows an example of the analysis carried out for five of the patient samples tested. Phosphorylation of PKC-δ was detected in both PEP005 responsive and PEP005 non-responsive patient samples. Densitometric analysis was performed on 28 patient samples (20 PEP005 responsive and 8 PEP005 non-responsive), measuring PKC-δ activation as a ratio of phosphorylated PKC-δ to the expression of total PKC-δ. Figures 3F-G show there was a significant activation of PKC-δ in response to PEP005 treatment in both PEP005 responsive and non-responsive primary AML samples. Therefore PKC-δ expression and activation following PEP005 treatment cannot differentiate between responsive and non-responsive patient samples.

### Gene expression in response to PEP005 treatment

**Figure 4:**
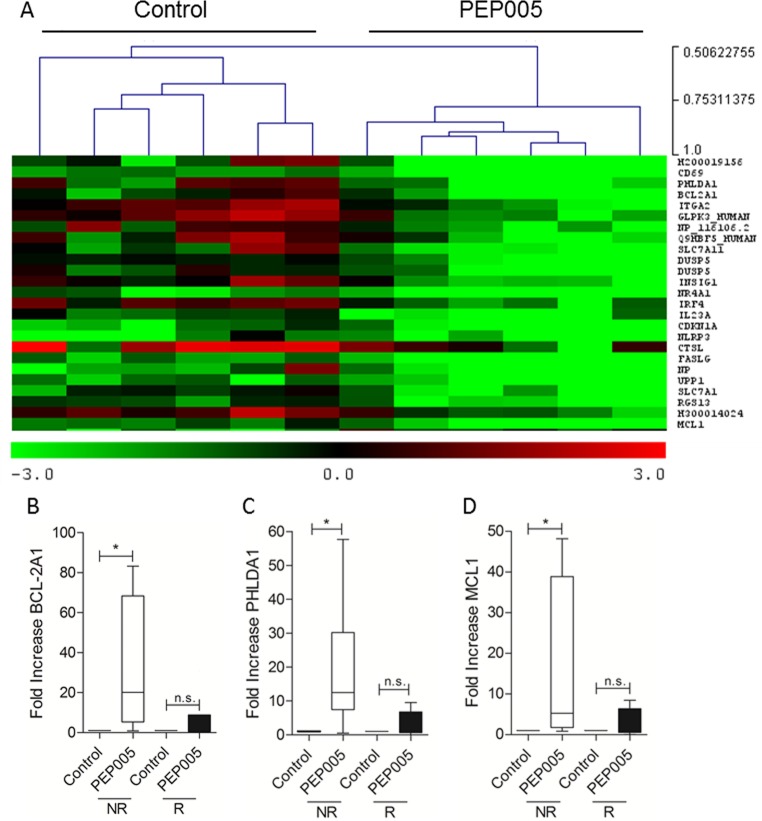
PEP005 treated non-responders show a distinct transcriptional profile A. **S**ignificance analysis of microarrays (SAM) gene lists were used to perform a hierarchical clustering for both the samples and genes. The heat map scale represents the gene value relative to a standard reference sample expressed as the log2 ratio of reference/sample; therefore green represents higher and red lower relative expression for each sample. B-D. Five non-responder and 4 responder AML blast samples were treated with 100 nM PEP005 and mRNA isolated after 6 hours and measured by quantitative PCR to confirm induction of Bcl-2A1 (B), PHLDA1 (C) and Mcl-1 (D). The horizontal bar shows the median value. * indicates p<0.05.

To try and determine patterns of gene expression that can differentiate responsive and non-responsive cells and identify the molecular basis of resistance to PEP005, we carried out whole genome expression microarray analysis in primary AML samples. 6 PEP005 responsive and 6 non-responsive primary AML samples were treated with either vehicle alone or 10 nM PEP005 for 6 hours. Samples were restricted to the AML 1 subtype to minimise variation. Cells were prepared for gene expression analysis using the Operon 37k whole genome array. Following normalisation, significance analysis of microarray (SAM) revealed no significant differences in gene expression between untreated PEP005-responsive and non-responsive samples. Furthermore, no differences in gene expression were found in the PEP005-responsive samples following PEP005 treatment. In contrast, when comparing PEP005 non-responsive cells, SAM analysis identified in total 134 genes (5% error) which showed a significant change in expression following PEP005 treatment. Using principle component analysis (PCA) and hierarchical cluster analysis (HCA), we were able to cluster separately the untreated samples from the PEP005-treated samples based on a mean expression level difference of 2.0 (Figure 4A). We then went on to use Predictive Analysis of Microarray (PAM) to identify alterations in which genes best predicted whether a sample was PEP005-treated or untreated. This resulted in a list of 25 genes which were ranked according to p-value from a paired T-Test (Table 2). 3 of the most predictive genes were all anti-apoptotic genes. As shown in Table 2, PHLDA-1, Bcl-2A1 and Mcl-1 were all significantly up-regulated in response to PEP005 treatment in the non-responsive samples.

**Table 2:** Predictive analysis of microarrays (PAM) was used to determine the change in expression of which genes best predicted whether a sample was a responder or a non-responder

Gene ID	Expression Difference	Control Mean	Control SD	PEP005 Mean	PEP005 SD	T value	P value
FASLG	2.391	−1.588	0.604	−3.979	1.125	9.109	0.00027
PHLDA1	3.123	0.100	1.324	−3.023	1.582	7.885	0.00053
CTSL	2.410	2.106	1.800	−0.304	1.805	7.813	0.00055
H200019156	4.483	−0.350	1.541	−4.833	1.997	6.944	0.00095
SLC7A1	2.166	−0.594	0.811	−2.759	1.229	6.842	0.00102
IRF4	2.531	0.836	0.606	−1.695	1.439	6.732	0.00110
MCL1	2.013	−1.121	0.498	−3.135	1.212	6.680	0.00114
ITGA2	2.933	1.088	0.710	−1.845	1.599	6.679	0.00114
IL23A	2.486	−0.800	0.540	−3.285	1.247	6.532	0.00126
GLPK3_HUMAN	2.847	1.241	0.792	−1.606	1.446	6.351	0.00143
NP	2.377	−1.422	1.577	−3.799	1.509	5.772	0.00219
BCL2A1	2.942	−0.400	1.122	−3.343	1.838	5.761	0.00221
UPP1	2.318	−1.766	1.018	−4.083	1.634	5.648	0.00242
CDKN1A	2.425	−1.781	1.020	−4.206	1.845	5.550	0.00261
NP_116106.2	2.742	0.270	1.038	−2.472	1.872	5.391	0.00296
SLC7A11	2.662	−0.179	1.405	−2.842	0.905	5.335	0.00310
CD69	3.438	−1.561	0.292	−4.998	1.664	5.209	0.00344
Q9HBF5_HUMAN	2.713	0.460	1.339	−2.253	1.945	5.069	0.00387
RGS13	2.084	−0.803	0.551	−2.887	1.210	4.855	0.00465
DUSP5	2.587	−0.419	0.742	−3.006	1.671	4.828	0.00477
DUSP5	2.591	−0.241	0.143	−2.831	1.378	4.698	0.00535
H300014024	2.030	1.046	0.682	−0.984	1.033	4.697	0.00535
NLRP3	2.413	−2.175	1.438	−4.588	2.434	4.684	0.00541
INSIG1	2.556	0.561	0.815	−1.995	1.215	4.651	0.00558
NR4A1	2.546	−1.867	1.047	−4.414	1.429	4.589	0.00590

### Validation of microarray data using RT-PCR

To validate the results obtained by microarray analysis, we measured gene expression of Bcl-2Al, Mcl-1 and PHLDA-1 relative to the housekeeping gene L27 using qPCR. A significant up-regulation in response to PEP005 treatment was seen for Bcl-2A1, Mcl-1 and PHLDA1 and was observed only in the resistant samples (Figure 4B), confirming the whole genome microarray data.

## DISCUSSION

We have previously shown that the novel small molecule PEP005 has potent anti-leukaemic effects, inducing apoptosis at nanomolar concentrations in leukaemic cell lines [13]. Here, we report that PEP005 also has potent anti-leukaemic effects against leukaemic cells isolated from patients in all AML classifications, inhibiting proliferation and inducing apoptosis at doses as low as 10 nM. We also conducted pre-clinical studies using PEP005 as a potential anti-AML reagent in two different human AML mouse xenograft models. In solid tumour xenografts of U937, systemic administration of PEP005 inhibited tumour growth significantly. In an alternative AML model, GFP-NB4 cells were intravenously transplanted into NOD/SCID and NOD/SCID/β2m^null^ mice. Although PEP005 treatment did not significantly increase the survival of the diseased mice, it did have a marked effect on tumour burden, particularly reducing GFP-NB4 cell infiltration in lymph nodes. Bearing in mind that this type of xenograft model is so aggressive that the average survival time of mice was approximately 30-40 days, at the time of PEP005 treatment (day 14), the disease was already well-established. Future studies should include optimized dosing schedule to better understand the anti-tumour effect.

Although PEP005 treatment led to apoptosis in the majority of patient samples tested, there were a small number that did not enter apoptosis in response to PEP005 treatment, even at the relatively high dose of 100 nM. We were therefore interested in trying to define a molecular response phenotype that would predict poor prognosis and resistance to treatment if PEP005 goes to clinical trial. We have previously shown in leukaemic cell lines, that the anti-leukaemic effects of PEP005 were dependent on the expression and activation of PKC-δ, an isoform of PKC which has long been associated with a pro-apoptotic function [17;18]. Despite the association between PEP005 responsiveness and the expression and activation of PKC-δ in leukaemic cell lines, we found that both PEP005 responsive and non-responsive primary AML blasts expressed high levels of PKC-δ. In addition, we found activation of PKC-δ in both patient sample groups in response to treatment with PEP005. Primary AML blasts thus do not appear to down regulate PKC-d and this isoenzyme was phosphorylated at Thr^505^ in response to PEP005. It is now well established that phosphorylation is essential for the regulation of PKC, enabling the enzymes to gain catalytic competence and correct intracellular localization [19]. However, in the case of PKC-δ, phosphorylation does not seem to be necessary to enable the enzyme to be a functional kinase. In particular, site directed mutagenesis studies have shown that phosphorylation at Thr^505^ is not essential for the activation of PKC-δ, unlike the corresponding threonine residues on PKC-α and PKC-βII [20]. Phosphorylation of PKC instead appears to be a priming event that locks PKC isoforms into a closed, stabilized, catalytically competent state.

It is possible that the translocation pattern of PKC-δ in response to PEP005 treatment may differ between the responsive and resistant samples. It is well documented that PKC requires translocation for its activation [21]. We have previously shown in leukaemic cell lines, that PEP005 treatment leads to a translocation of PKC-δ to the nucleus, a pattern of translocation particularly associated with apoptosis [17;18;22], and this pattern of PKC-δ translocation in response to PEP005 has also been shown in CHO-K1 cells transfected with GFP-tagged PKC-δ [23]. Future studies should investigate whether PKC-d translocation differed between resistant and sensitive cells.

To further elucidate the mechanism of resistance to PEP005, we carried out whole genome expression microarray analysis. We found approximately 134 genes that showed a significant difference in expression levels following PEP005 treatment in the resistant samples. PAM revealed that of these genes, some of the 25 most predictive were genes that encoded for proteins with known anti-apoptotic function. These included Bcl-2A1, Mcl-1 and PHLDA1, the increased expression of which was confirmed using qPCR. Numerous studies have demonstrated overexpression of Bcl-2A1 in human leukaemia's including Acute Lymphoblastic Leukaemia (ALL) [24;25] and AML [25;26]. In addition, Bcl-2A1 has already been suggested to be involved in promoting drug resistance in cancer cell lines. In particular, the over-expression of Bcl-2A1 has been shown to mediate the resistance to etoposide [27] and staurosporine [28]. Bcl-2A1 has also been shown to be up-regulated in response to GM-CSF treatment in the AML cell line TF-1, conferring resistance to TNFα mediated apoptosis [29]. Moreover, the expression of Bcl-2A1 has been shown to correlate with resistance to chemotherapy *in vivo*, in Chronic Lymphocytic Leukaemia (CLL) [30]. Similarly, Mcl-1 has been shown to be expressed at high levels in the leukaemic stem cell fraction of AML cells when compared to normal haematopoietic stem cells [31] and Mcl-1 over-expression has been shown to have a role in resistance of leukaemic cells to Flt-3 inhibitors [32]. The increased expression of the genes in response to PEP005 may explain why these particular samples are resistant to the ability of the compound to induce apoptosis as well as providing a simple way to test for potential responsiveness to the compound.

In summary, we have demonstrated that the novel plant derived compound PEP005 has potent anti-leukaemic effects both *in vitro* and *in vivo* and have shown that certain non-responsive leukaemic cells are rendered resistant to apoptosis induction by up-regulating key anti-apoptotic genes. The latter may represent a useful diagnostic marker of responsiveness to PEP005 if the compound progresses to clinical trial.

## MATERIALS AND METHODS

Reagents were purchased from Sigma-Aldrich unless otherwise stated.

### PEP005 preparation

PEP005 was supplied as a 98.5% pure preparation by Peplin Ltd, (Brisbane, Australia) as a dry pellet and was made up to a stock of 10 mg/ml in dimethyl sulfoxide (DMSO). Stocks were diluted in RPMI-1640 media supplemented with 10% heat inactivated (HI) fetal calf serum (FCS, Sera Laboratories International) (vol/vol), streptomycin (100 ug/ml), penicillin (100 U/ml) and 2 mM L-glutamine.

### Purification of Peripheral Blood Mononuclear Cells (PBMCs)

The study was approved by the local Research Ethics Committee and all samples were collected after written informed consent. Leukaemic PBMCs were isolated from the peripheral blood of AML patients with at least 80% leukaemic blasts amongst leucocytes using Ficoll (GE Healthcare) density centrifugation. The percentage of leukaemic blasts in isolated samples exceeded 95% for all patients as judged by light microscopy.

### Culture and treatment of isolated cells

To examine the effects of PEP005 on leukaemic cell survival and proliferation, cells were treated with a vehicle control or PEP005 (2 nM-20 μM).

### Detection of apoptosis

Cells were stained with Annexin-V and Propidium Iodide using a commercially available apoptosis assay kit according to the manufacturer's instructions (Apotest, Nexins Research). 10,000 events were analysed on a FACSCalibur cytometer (Becton Dickinson) and data analysed using WinMDI software.

### Measurement of proliferation using 3H-thymidine incorporation

AML blasts were resuspended at a concentration of 2 × 10^6^/ml in stem span medium (Stem Cell Technologies) supplemented with 20 ng/ml of recombinant human Flt-3 ligand, stem cell factor (SCF) and granulocyte-macrophage colony-stimulating factor (GM-CSF) (all from Peprotech Ltd), and 100 μg/ml gentamicin. 2 × 10^5^ cells were treated with either medium alone or PEP005 (1nM-1μM) and cultured at 37°C/5% CO_2_ for 7 days. Post culture, ^3^H-thymidine (Amersham Biosciences) was added in 20 μl of 0.9% NaCl solution and 18 hours later, cells harvested using a Skatron Micro96 harvester onto filter mats (Cox Scientific). Filter mats were left to air dry and ^3^H-thymidine incorporation was measured using a liquid scintillation counter. Results are expressed as counts per minute (CPM).

### Animals

Male and female NOD-SCID mice (Table 2) and NOD/LtSz-*Prkdc^scid^/B2m^null^* mice (Figure 3, abbreviated as NOD/SCID/β2m^null^), originally obtained from Dr. Leonard Schultz, (Jackson Laboratories, Bar Harbor, Maine, USA) were used for the GFP-NB4 leukaemia model. Nude mice were used for the U937 subcutaneous leukaemia model. All experiments were approved by The Norwegian or German Animal Research Authority and conducted according to The European Convention for the Protection of Vertebrates Used for Scientific Purposes.

### U937 subcutaneous leukaemia model

6-8 week old nude mice were subcutaneously injected with 5 × 10^6^ U937 cells. 14 days post inoculation (normalized as day 0), mice (8 mice per group) received tail vein injections of different doses (40, 60 and 80mg/kg/d) of PEP005-PEG400 (provided by Peplin Ltd.) or vehicle control 3 times a week for two weeks. Tumor volume was determined by a two dimensional measurement with calliper on the day of randomization and then twice weekly. The relative volume of an individual tumor on day X (RTVx) is calculated by dividing the absolute volume [mm^3^] of the tumor on day X (Tx) by the absolute volume of the same tumor on the day of randomization, multiplied by 100 (RTVx=Tx/T0*100). Mice were sacrificed when individual tumor volume exceeded 2000mm^3^, body weight loss exceeded 30% and/or when a severe impairment of general condition was observed.

### GFP-NB4 leukaemia model

6-8 week old NOD/SCID and NOD/SCID/β2m^null^ mice were irradiated from a photon radiation source (BCC Dynaray CH4, 4 megavolt photon irradiation source, with a sub-lethal dose of 2.5 Gy (60 cGy/min)) 24 hours prior to transplantation. NB4-GFP cells (10^7^) were injected via the dorsal tail vein. 2 weeks after GFP-NB4 inoculation, mice (6 mice per group) received tail vein injections of 50 μg/kg PEP005-PEG400 or vehicle control once daily for 5 consecutive days. Recipient mice were monitored by weighing every second day and imaging every 3 days, and sacrificed following institutional guidelines when moribund, as defined by weight loss, lethargy and/or paralysis.

In order to directly compare leukaemic infiltration and tumour burden between control and treated mice, different tissues were collected after mice were sacrificed. Tissues were homogenised and filtered through a 40 μm cell strainer (BD Biosciences). Red blood cells were lysed using lysing solution (150mM NH_4_Cl, 10mM KHCO_3_ and 20mM EDTA). Samples with acquisitions of 10,000 cells were subsequently run on a FACS Calibur flow cytometer (BD Biosciences) and gated against GFP^+^ cells.

### Time-Domain GFP Fluorescence Imaging

Anaesthetised mice (1% isofluorane), were depilated and moved to the heated translational stage of an eXpore Optix™ (ART/GE Healthcare) configured for GFP imaging experiments as previously described [14]. The integration time and laser power for animals was optimised per animal prior to scanning, with a raster scan interval of 1 mm. Whole body images of leukaemic mice were taken prior to leukaemic inoculation and used as representative background auto fluorescence, per animal. GFP fluorescence images were generated using Optiview™ software (ART/GE Healthcare) gating for fluorescence lifetime of GFP at 2.7 ns as previously described [14].

### SDS-PAGE and western blotting

Expression and activation of PKC-δ were determined by western blotting of cell extracts using antibodies to total PKC-δ (Santa Cruz) as well as PKC-δ phosphorylated at threonine 505 (Cell Signalling Technologies). Total cell lysates were obtained by resuspending the cells in SDS sample buffer (0.5 M Tris pH 6.8, 10% β-mercaptoethanol, 10% SDS, 20% Glycerol, 0.1% bromophenol blue) and heated for 10 minutes at 100°C. Lysates were loaded onto a 12% SDS-polyacrylamide gel (SDS-PAGE). Proteins were subsequently transferred to 0.45 micron polyvinylidene difluoride membrane (Flowgen). Blots were probed with the relevant primary antibodies and secondary antibodies conjugated to horse radish peroxidase (Dako Ltd) after which, proteins were visualised using enhanced chemiluminescence (Amersham Pharmacia) and autoradiography against X-ray film (Kodak). Equal loading of proteins was assessed by probing blots for β-actin.

### Gene expression microarray analysis

RNA was extracted using the Qiagen RNAeasy kit according to manufacturer's instructions (Qiagen). Reverse transcription of the RNA was achieved by adding 5μg of RNA to 1μl oligo(dT)_12-18_ primer (Invitrogen). This was then heated to 65^0^C for 10 minutes, and cooled on ice before addition of 8μl of 1^st^ strand buffer, 4μl 0.1M DTT (Invitrogen), and 2μl Superscript II 200U/μl (Invitrogen) as well as 8μl 10mM dNTPs (Invitrogen). The reaction was then kept at 42^0^C for 2 hours. cDNA was extracted using a PCR Purification Kit (Machery-Nagel) as per the kit instructions. Cy3-dCTP and Cy5-dCTP labelling was performed using 500 ng of sample cDNA and the labelled cDNA was purified using a PCR Purification Kit. Labelled cDNA was then added to a microarray consisting of the Human Genome Array Ready Oligo Set version 4.0 (Operon). Data were acquired using a ScanArray Gx Plus (PerkinElmer). All samples were run in parallel to a Universal Human Reference RNA sample. Hybridizations were carried out within Corning Hybridization Chambers overnight, using Lifter Slips (VWR International). Data normalization was performed using the Gene Expression Pattern Analysis Suite (http://www.gepas.org) [15]. Using log-normalized ratios, the TIGR Multiexperiment Viewer version 4.0 was used to perform significance analysis of microarrays. Hierarchical clustering and principal component analysis were performed on the resultant gene list.

### mRNA quantification by quantitative real time PCR (qPCR)

B-cell lymphoma 2-related protein A1 (Bcl-2A1), myeloid cell leukaemia sequence 1 (Mcl-1), Pleckstrin homology-like domain family A member 1 (PHLDA1) and L-27 mRNA were analysed by qPCR using Taqman PCR probes. Bcl-2A1, Mcl-1 and PHLDA-1 FAM-labelled primers, and L-27 VIC-labelled primers were synthesised by Eurogentec. Samples were amplified using the Stratagene MX3000P qPCR machine and analysed using MXPro software (Agilent Technologies).

### Statistical analysis

Non-parametric statistical analysis was used that included the Mann Whitney U test, the Wilcoxon Signed Rank Test and the Kruskal-Wallis test. When analysing data from a normal (Gausian) distribution, parametric tests such as t-test, paired t-test and one way ANOVA were used. All statistical analysis was carried out using version 4 of the GraphPad Prism software (GraphPad software limited). Results were considered significant at p<0.05.

## SUPPLEMENTARY TABLE


