# Supplementary material for: Up-regulation of anti-apoptotic genes confers resistance to the novel anti-leukaemic compound PEP005 in primary AML cells
Source: Oncoscience. 2014 Aug 6;1(8):529–39. doi: 10.18632/oncoscience.71 (PMC4278328; doi:10.18632/oncoscience.71)
Supplement: Supplementary file 1 [file oncoscience-01-0529-s001.pdf]

Up-regulation of anti-apoptotic genes confers resistance to the novel anti-leukaemic compound PEP005 in primary AML cells

Supplementary Table 1: Patient Characteristics

Patient	Sex	Age	FAB	Previous	Flt3	CD11c	CD13	CD14	CD15	CD33	CD34	CD45	HLA-DR	Cytogenetics	NPM-1	PEP005 Response
1	F	81	M1	MDS	wt	46	58	1	3	61	73	95	64	Normal	wt	NR
2	F	46	M1		ITD	37	99	1	5	67	35	nt	76	Normal	ins	NR
3	M	68	M1	MDS	ITD	nt	18	5	nt	59	1	nt	4	nt	ins	NR
4	M	69	M1	MDS	wt	72	99	nt	45	99	97	100	97	nt	wt	NR
5	M	63	M1		wt	nt	95	7	8	11	84	100	78	t(3;18)	wt	NR
6	M	52	M1	Relapse	wt	35	63	3	70	27	94	100	82	Multiple	nt	NR
7	M	79	M4		ITD	nt	13	7	32	66	2	nt	nt	nt	nt	NR
8	F	66	M1		wt	nt	15	nt	5	97	1	nt	1	Normal	ins	LR
9	M	72	M5		ITD	96	31	40	69	99	1	90	63	Normal	ins	LR
10	F	58	M2		ITD	57	93	1	nt	98	56	99	78	Normal	ins	LR
11	F	36	M4		wt	77	61	46	47	63	22	100	85	Normal	nt	LR
12	M	29	M4	Relapse	ITD	43	92	5	24	96	55	100	73	Normal	ins	LR
13	M	67	M0		ITD	17	6	1	8	9	99	9	99	del(5)	wt	HR
14	M	62	M4		nt	16	79	1	3	33	98	96	94	Trisomy 8	wt	HR
15	M	29	M4	Relapse	ITD	43	92	5	24	96	55	100	73	Normal	ins	HR
16	M	80	M1		nt	37	85	2	11	76	85	98	92	Multiple	nt	HR
17	M	72	M1		wt	52	85	2	34	60	84	97	91	pluss-8	wt	HR
18	M	57	M2		wt	33	100	1	54	30	76	100	96	Multiple	nt	HR
19	F	79	M4		G835	36	60	15	86	95	40	97	52	nt	wt	HR
20	F	77	M1		nt	2	72	2	66	3	87	100	70	nt	wt	HR
21	F	68	M1	Relapse	ITD	23	100	1	35	28	98	99	98	Normal	wt	HR
22	M	64	M1	MDS	ITD	29	57	4	86	95	40	96	52	Multiple	nt	HR
23	M	63	M1		wt	39	82	32	nt	41	68	78	92	Normal	nt	HR
24	M	72	M1		wt	nt	100	nt	nt	90	99	99	84	inv(16)	wt	HR
25	M	52	M2	Relapse	nt	45	17	1	54	71	1	96	34	nt	wt	HR
26	F	45	M1		ITD, D835	nt	42	2	3	98	6	100	2	Normal	ins	HR
27	F	75	M4		ITD	91	98	9	55	75	76	90	67	Normal	wt	HR

28	M	82	M5		wt	90	19	20	100	98	1	98	46	Mangler-Y	wt	HR
29	F	56	M2		wt	78	83	1	13	91	2	100	49	nt	wt	HR
30	F	45	M4		wt	nt	90	1	24	98	1	98	1	Normal	ins	HR
31	M	48	M4		wt	29	97	2	48	75	78	100	95	inv(16)	wt	HR
32	F	61	M5		wt	99	43	91	82	99	1	100	96	Normal	ins	HR
33	F	67	M4		nt	89	80	3	38	95	79	100	48	t(16;16), +22	nt	HR

Patient characteristics, AML subtype (FAB classification), membrane expression (shown as % positive cells), cytogenetics, Flt3 mutation status, NPM mutations status and PEP005 responsiveness. Cytogenetic abnormalities were classified according to MRC guidelines[33]. wt, wild type; nt, not tested: ITD, internal tandem duplication; MDS, myelodysplastic syndrome; Flt3, fms-related tyrosine kinase 3; NPM, nucleophosmin; NR, non-responder; LR, low-responder; HR, high-responder.
